# Photopolymerization of Limonene Dioxide and Vegetable Oils as Biobased 3D-Printing Stereolithographic Formulation

**DOI:** 10.3390/polym16070965

**Published:** 2024-04-02

**Authors:** Mégane Clerget, Eric Gagnon, Jerome P. Claverie

**Affiliations:** 1 Chemistry Department, Université de Sherbrooke, 2500 Boulevard de l’Université, Sherbrooke, QC J1K 2R1, Canada; 2 Soprema Canada, 1688 rue Jean Berchmans Michaud, Drummondville, QC J2C 8E9, Canada

**Keywords:** epoxy, 3D printing, stereolithography, limonene dioxide, biobased formulation

## Abstract

Epoxidized vegetable oils and limonene dioxide, a bis-epoxide derived from the terpene limonene, are photo-copolymerized to yield highly crosslinked networks with high conversion of all epoxide groups at ambient temperature. However, the slow polymerization of such biobased formulation polymerizes is not compatible for a use in a commercial SLA 3D printer. Adding an acrylated epoxidized vegetable oil to the bis-epoxide leads to a decrease of curing time and an increase in LDO conversion to polymer. For example, in a 60:40 wt:wt mixture of LDO and epoxidized soybean oil, the conversions of both exocyclic and endocyclic epoxide groups of LDO are ≥95%. These formulations were successfully used in SLA 3D printers, leading to generation of hard and dry complex objects using biobased formulations.

## 1. Introduction

Countless technologies rely on synthetic polymers which are derived from fossil resources [1]. Up to 20% of polymeric products are thermosetting polymers, and among those, epoxy polymers occupy a predominant position [2]. The vast majority of such epoxy polymers are based on the bisphenol A diglycidyl ether monomer (BADGE) [3] which originates from benzene, propene and epichlorhydrine. Although epichlorhydrine can be biosourced [2] benzene and propene remain major petrochemicals. Thus, the quest for biobased epoxy polymers, and more generally for biobased thermosetting polymers free of bisphenols, is currently receiving considerable attention [2,4].

**Figure 1:**
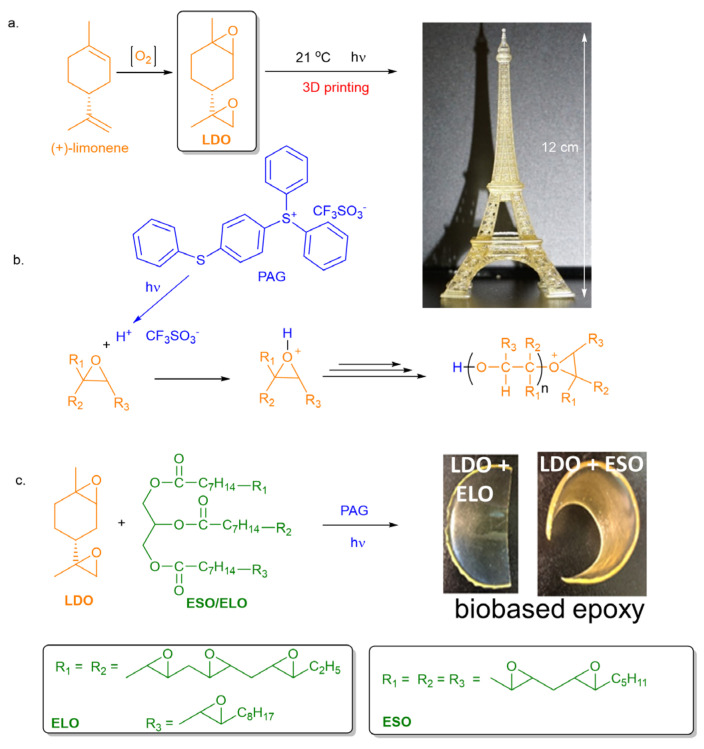
Cationic polymerization of limonene dioxide (LDO). (**a**) Structure of LDO and 3D-printed object printed with LDO-based formulation. (**b**) Mechanism of an epoxide cationic polymerization and structure of the sulfonium photoacid generator (PAG) used in this study. (**c**) Polymer obtained upon the cationic photopolymerization of LDO formulated with epoxidized soybean oil (ESO) or epoxidized linseed oil (ELO).

Among biobased alternatives to BADGE, limonene dioxide, LDO, seems particularly advantageous. Indeed, this liquid monomer can be easily prepared by the catalytic oxidation of (+)-limonene in a one-step process using molecular oxygen as an oxidant (Figure 1a) [5]. Vast amounts of this terpene can be directly extracted from orange and lemon peels [6,7]. However, the preparation of thermosetting polymers from LDO presents inherent difficulties. Indeed, LDO derived from (+)-limonene is in fact a mixture of four isomers, two of which are less reactive upon ring-opening by nucleophilic addition [8]. Thus, epoxy polymers obtained by reaction of diamines with LDO are only fully crosslinked when the two *trans* isomers which are the most reactive monomers are used [9]. Mija et al. recently solved this problem by using biobased glutaric anhydride as hardener instead of diamines [10]. However, temperatures as high as 210 °C were necessary to fully crosslink the polymer, which is indicative of the low reactivity of LDO. Using lanthanide-based catalysts, we recently demonstrated that fully crosslinked epoxy based thermosets based on LDO could be prepared at lower temperatures (120–140 °C) [11]. However, such temperatures are still too high for many applications since thermosetting polymers are often prepared on site and without the assistance of any specific equipment. From a green chemistry point of view, higher temperature entails greater energy input, which is also undesirable.

In response to the challenges of thermal polymerization of LDO, the cationic photopolymerization process is particularly appealing due to its reduced energy consumption and low VOC (volatile organic compounds) emission, while allowing spatial and temporal control which opens the door to 3D-printing processes (Figure 1b) [12,13].

In this work, we first provide insights on the cationic photopolymerization of LDO initiated by diaryliodonium and triarylsulfonium photoinitiators [14]. In such a process, the photoactivation of the initiator leads to the formation of a strong acid which then forms an oxonium. This oxonium is the active species of the cationic polymerization (Figure 1b). Due to its high rigidity, the cationic photopolymerization of LDO leads to the formation of a polymer with a high glass transition temperature, T_g_. The polymerization being performed at room temperature, the polymerization stops before reaching complete conversion as the diffusion of both monomer and polymer chains is sluggish in a vitreous matrix (vitrification phenomenon). To address this issue, we have combined LDO with another biobased epoxy monomer, either epoxidized soybean oil (ESO) or epoxidized linseed oil (ELO, Figure 1c) [15,16]. Due to their flexible backbones, these monomers, when combined to LDO, are able to form a highly crosslinked thermoset polymer, thus demonstrating that biobased formulations based on LDO can be obtained by photopolymerization.

Encouraged by this first series of results, we evaluated whether these formulations could be used in a stereolithographic 3D-printing process (SLA). The SLA process is an additive manufacturing process whereby a reactive thermosetting formulation is solidified upon layer-by-layer exposure to a laser light of suitable wavelength [17]. The SLA process offers many advantages including a short manufacturing cycle, the capability of fabricating complex forms that cannot fabricated by other means, and a reduced material waste [18]. SLA formulations contain two types of functionalities, one which is reactive via a cationic mechanism, and the other one which reacts via a radical mechanism. This dual-curing procedure has been reported in the past [19,20,21] and is actually employed in commercial SLA 3D-printing formulations [22]. By adding as little as 15% of the latter one to the cationic LDO-based formulation, we were able to design a monomer composition which was easily 3D-printed in current SLA 3D printers (Figure 1). To our knowledge, such compositions constitute the first example of mostly biobased formulation for SLA 3D printers.

## 2. Materials and Methods

Limonene dioxide (LDO) was purchased from Symrise (Holzminden, Germany). Epoxidized soybean oil (ESO) and epoxidized linseed oil (ELO) were obtained from Brenntag (Lachine, QC, Canada). Trimethylolpropane triacrylate (TMPTA), phenylbis(2,4,6-trimethylbenzoyl) phosphine oxide (BAPO), acrylated epoxidized soybean oil (AESO), bis(4-methylphenyl) iodonium hexafluorophosphate (4-Phenylthiophenyl) diphenylsulfonium triflate (PAG) were purchased from Sigma-Aldrich (Mississauga, ON, Canada) and used as received. Irradiation was realized with a mercury lamp Dymax 2000 (Torringto, CT, USA) flood lamp. The lamp was calibrated with a spectroradiometer and was found to deliver an irradiance of 49 mW/cm^2^ centered on the wavelength λ = 300 nm. The 3D printer was a Voxelab Polaris 2K Color LCD Resin 3D Printer (Jinhua City, China) equipped with a 405 nm lamp. It has a nominal resolution of 0.047 mm, and a print speed which can be adjusted between 10 to 50 mm/h.

### 2.1. Cationic Photopolymerization

Formulations were prepared by weighing LDO and the photoacid generator in a vial (see Table 1 for proportions). Those components were mixed for 2 min in an ultrasonic bath. Epoxidized vegetable oil was then added and homogenized using a vortex stirrer for 1 min. The solution was poured in an aluminium dish to form a 1 mm deep layer which was irradiated for one minute under the flood lamp at room temperature. The light was then turned off and the polymerization was left to proceed for several hours. Drying time (ASTM D5895) [23] was assessed on a Beck Koller drying-time recorder.

### 2.2. 3D Printing Application

In a vial, the PAG and BAPO were mixed in LDO for two minutes in an ultrasonic bath. Both PAG and BAPO were added so that they each account for 0.5 wt% of the total sample mass (LDO, AESO + TMPTA). Then TMPTA and AESO were added and homogenized using a vortex stirrer for 1 min. This solution was then poured into the 3D printer and the printing was started. The thickness of each layer was set as 0.1 mm with an exposure time of 20 s. The obtained 3D structure was washed with isopropanol.

### 2.3. Swelling Test

Squares of solid epoxy material of 1 cm dimension and 2 mm thickness were immersed in methanol for three days. After three days of immersion, the samples of known initial mass (w_1_) were removed from the solvent, patted dry to absorb the excess solvent and weighed (w_2_). The swelling percentage, SP%, was calculated using the formula SP% = 100·(w_2_ − w_1_)/w_1_

### 2.4. Conversion Measurement by Fourier Transform InfraRed (FTIR) Spectroscopy

**Figure 2:**
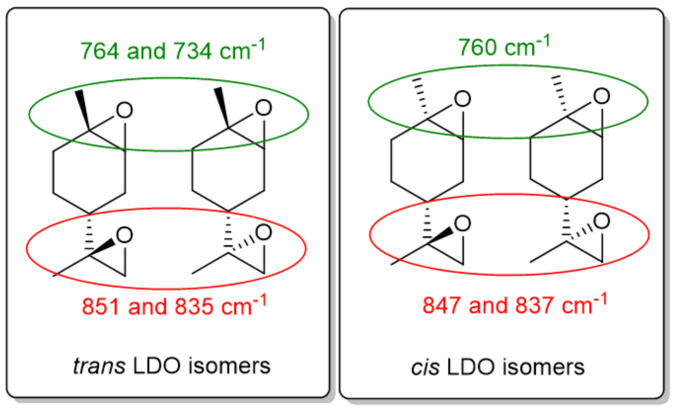
*Cis* and *trans* isomers of LDO, and attribution of the epoxide FTIR signals.

FTIR spectra were acquired on an Agilent Cary 630 (Santa Clara, CA, USA) equipped with an ATR accessory. Each spectrum was the average of 64 scans collected between 600 and 4000 cm^−1^ with a resolution of 2 cm^−1^. The conversion of LDO was measured by following the bands at 760 cm^−1^ and 850 cm^−1^ using the attribution found in reference [9] (Figure 2) while the epoxy band at 830 cm^−1^ was used for ESO or ELO [24]. In the 3D-printing process, the characteristic vinyl absorption peak of acrylates was monitored at 1630 cm^−1^ in order to measure the acrylate conversion. For the sake of comparison, the FTIR spectra were normalized using the band at 2950 cm^−1^ which corresponds to the CH vibrations. As the photopolymerization does not affect the concentration of CH bonds, the normalization can be used to compare samples in between them.

### 2.5. Dynamic Mechanical Temperature Measurements (DMTA)

The loss modulus, E″, storage modulus, E′ and mechanical loss tangent, tan δ were evaluated by DMTA in torsion mode using a frequency of 1 Hz on a Physica MCR 301 Anton Paar (Ashland, VA, USA) instrument fitted with a LN cooling system. Cured samples were cut in strips of size 50 mm × 10 mm × 1 mm and analyzed between −30 to 100 °C with a heating rate of 2 °C/min, for the cationic polymerization, and between −90 °C to 100 °C with a heating rate of 2 °C/min for the 3D printed samples. The glass transition temperature, T_g_, was taken at the maximum value of tan δ.

### 2.6. Differential Scanning Calorimetry (DSC)

Differential scanning calorimetry (DSC) measurements were performed on a DSC25 from TA Instruments (New Castle, DE, USA). Approximately 10 mg of sample were inserted in an aluminium pan. Two heating–cooling cycles were applied using a 10 °C/min ramp. The sample was heated from 0 °C to 150 °C, cooled to −50 °C and then heated to 150 °C. For the 3D printed samples, the sample was heated from 0 °C to 200 °C, cooled to −90 °C and then heated to 200 °C. The glass transition temperature (T_g_) was determined from the second heating curve.

## 3. Results

### 3.1. Polymerization of LDO

**Figure 3:**
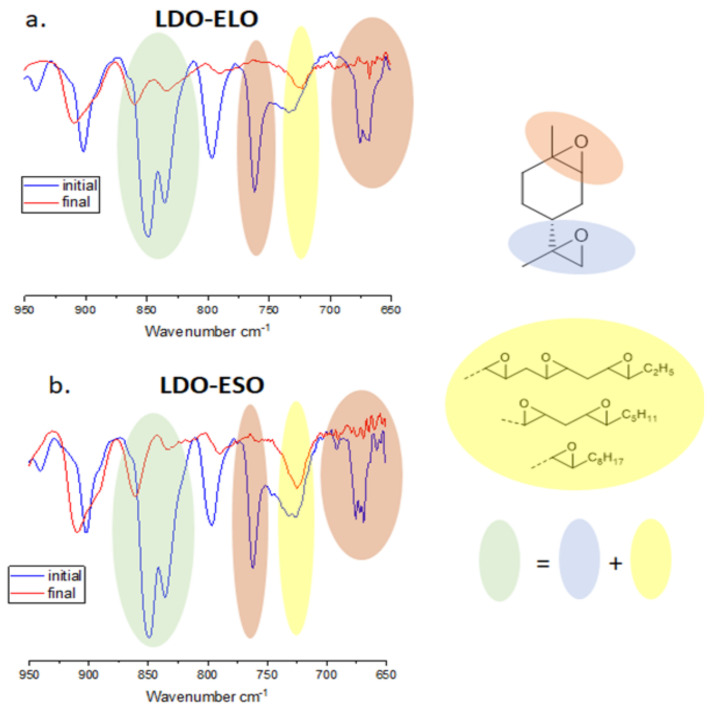
FTIR spectrum of (**a**) LDO-ELO and (**b**) LDO-ESO (50 wt%) mixtures before and after photocationic polymerization (PAG = 1 wt%, 1 min irradiation, 60% relative humidity). The attribution of each band is color-coded. The band at 820–850 cm^−1^ (green) is the overlap of the exocyclic LDO epoxide and vegetable oil epoxides. The spectra were normalized using the band at 2950 cm^−1^ which corresponds to CH vibrations.

In our experiments, the photopolymerization of LDO was initiated by a sulfonium triflate PAG (Figure 1b). This PAG has a maximum absorption at λ_max_ = 298 nm. At this wavelength, LDO is essentially transparent. The LDO conversion was followed by FTIR spectroscopy. LDO is in fact a mixture of four isomers (Figure 2) and the wavenumbers of the symmetric epoxide ring deformation vibration can be attributed for both endocyclic and exocyclic epoxides (see Figure S1 for FTIR spectra of *cis* and *trans* monomers). During the polymerization, these characteristic epoxy bands disappear and, concomitantly, a band at around 3450 cm^−1^ appears. This band corresponds to the OH group which is formed upon epoxide ring-opening (Figure 3 and Figure S2). The conversion of each epoxide, which is related to the absorbance of each band, was calculated using:

(1) $$A\left(t\right)=\left(1-\frac{\frac{A}{A^{0}}}{\frac{A_{\mathrm{ref}}}{A_{\mathrm{ref}}^{0}}}\;\right)\times 100$$

where A and A^0^ are the absorbance of the peak at time t and at initial time respectively, and A_ref_ and A^0^_ref_ are the absorbance of a reference band at 2927 cm^−1^ which corresponds to the aliphatic CH vibrations (their concentration remains constant during the polymerization). The normalization by a reference absorbance is necessary to compensate for the change in thickness of the sample during polymerization. In a typical experiment, the LDO/PAG mixture containing 0.1 wt% PAG was irradiated for 1 min at room temperature and was then left to polymerize until dried. Under such conditions, the conversion of endocyclic epoxides (~760 cm^−1^, Figure 2) is greater than 90% after 2 h at room temperature. By contrast, the exocyclic epoxides (~850 cm^−1^) are only 50% converted (Figure S3 and Table 1). Using longer polymerization times does not result in an increase of the conversion. This phenomenon can be explained by the vitrification of the thermoset polymer resulting from the high T_g_ of the LDO-based thermoset [9]. In fact, the resulting polymer is very brittle and stress cracks are observed along the sample (Figure S4). Remarkably, using pure *cis*-LDO or pure *trans*-LDO isomers leads to identical results in terms of conversion. Thus, unlike thermal polymerization, the cationic photopolymerization is insensitive to the stereochemistry of LDO. Based on this result, LDO was used as a mixture of isomers (54% *cis*, 46% *trans*, as shown by quantitative ^13^C NMR using attributions shown in reference [9]) for all further experiments.

Although the polymerization of the exocyclic epoxide is not complete, the polymer can be considered dried, as measured by the ASTM D5895 standard test which records the time necessary for the sample to become hard. When the PAG concentration was increased from 0.1 to 0.5 wt% and irradiated for 1 min, the drying time was found to decrease from 90 to 60 min. The drying time was also found to decrease with longer irradiation time. For example, after 1, 5 or 10 min irradiation, the sample (containing 0.1 wt% PAG) dried in respectively 90, 70 and 10 min. Furthermore, the drying time was also affected by the relative humidity. At 30%, 60% and 80% relative humidity, the drying time was, respectively, 155, 90 and 55 min. The decrease of drying time with increasing humidity can be explained by the fact that H_2_O or any protic compound will act as an efficient transfer agent for the reactive species (oxonium of Figure 1b). As the polymerization progresses, the viscosity increases and the attack of the oxonium by an unreacted epoxide slows down. Upon reaction of the oxonium with water, the proton can be transferred to a more accessible epoxide group, resulting in a faster polymerization. Although the drying time can be tuned by changing the irradiation time, PAG concentration and relative humidity, in all cases the conversion of the exocyclic epoxide was found to cap at 60% (Figures S3, S5 and S6). Furthermore, the polymer is always cracked. By analyzing the sample by DSC, no T_g_ could be detected at temperatures as high as 120 °C, whereas the sample remains hard at that temperature. Thus, although the photocationic polymerization of LDO proceeds at room temperature, it is limited by a vitrification phenomenon: in other words, the T_g_ of the polymer is too high to allow complete crosslinking. The fact that only endocyclic epoxides are completely converted can be explained by the fact that trisubstituted (more electron-rich) epoxides are more reactive than disubstituted ones. Based on these results, all subsequent copolymerizations were performed using a 0.1 wt% PAG concentration, a 1-min irradiation time and a relative humidity of 60%.

### 3.2. Copolymerization of LDO with Epoxidized Vegetable Oils

To achieve a higher conversion and prevent vitrification, biobased epoxidized vegetable oils were considered as additional monomers (Figure 1c). These compounds are biobased epoxy monomers that lead to low T_g_ polymers [15,16,25]. Thus, using only epoxidized vegetable oils such as ESO and ELO under photocationic polymerization conditions yielded very soft and tacky materials that cannot be used for most usages, it was found that combining them with LDO in various ratio under the same photopolymerization conditions gave flexible crack-free solid samples (Figure 1).

The conversion of both endocyclic ”nd e’ocyclic LDO epoxides can be monitored by FTIR spectroscopy. However, the analysis is complicated by the fact that the vibrations of the vegetable oil epoxides and of the exocyclic LDO epoxide overlap at 820–850 cm^−1^ (Figure 3). The conversion of the vegetable epoxides was measured using the characteristic vibration at 720 cm^−1^. This peak is very close to the endocyclic LDO vibration at 750 cm^−1^, and therefore these two peaks were first deconvoluted in two Lorentzian before using Equation (1). Once known the conversion of the vegetable oil, it was possible to estimate the conversion of the exocyclic LDO epoxide by subtracting the spectrum of the unreacted vegetable oil to the spectrum of the polymerized mixture, thus giving a calculated band at 820–850 cm^−1^ that only took into account the LDO vibrations.

By combining ESO or ELO to LDO, the conversion of the exocyclic LDO monomer is significantly increased (Table 1 and Figure S7). In fact, in most cases, LDO is entirely polymerized. This high conversion as well as the flexibility of the polymerized sample are indicative that the vitrification phenomenon does not occur in contrast to pure LDO samples (see below for T_g_ determination). The conversion of the vegetable oil is, however, not quantitative. Such a result can be explained by the fact that these vegetables oils contain epoxide groups that are very close to each other; particularly in the case of ELO (see Figure 1c). For example, it is unlikely that the three adjacent epoxides can all polymerize, simply due to steric constrains. In fact, the number of functionalities measured experimentally for these oils is lower than the expected value (5.2 for ELO, and 3.5 for ESO) [15]. Although the conversions of the ESO or ELO epoxides are not quantitative, it will be seen below that no ESO or ELO remains unreacted; in other word, the polymers are devoid of soluble fractions.

Despite the high LDO conversion, it is found that the drying times of ESO or ELO-containing formulations are significantly longer than for pure LDO formulations (Table 1). In LDO, the most reactive epoxide is the tri-substituted (endocyclic) one. In ESO and ELO, all epoxides are disubstituted and therefore less reactive for the cationic polymerization. As a result, the polymerization proceeds more slowly. However, in all cases, the polymerization proceeds to completion (dryness) in less than 24 h at room temperature.

**Figure 4:**
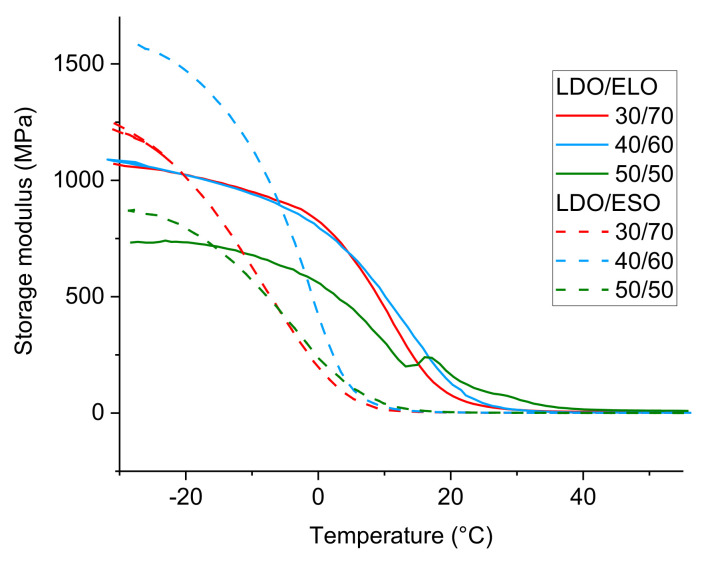
DMTA curves of LDO/ELO and LDO/ESO polymers (1% of PAG, irradiation for 1 min, 60% relative humidity).

The pure LDO sample was found to be soluble in methanol indicating that the absence of crosslinks. By contrast, copolymers of LDO and ESO withstood dissolution in methanol (Table 2), water or hexane, indicating that they are crosslinked. As shown in Table 2, when LDO is used with 40% of epoxidized oil, the swelling ratio in methanol is high, which means that the crosslink density is lower. At identical composition, swelling results for ESO are higher than ELO, because of the lower number of functionalities of ESO. By adding a higher concentration of ESO or ELO, swelling decreases, which can be explained by a higher crosslink density stemming from the high functionality of these monomers. In all cases, a fully cured (dried) film was obtained, which allowed the characterization of the materials by DMTA (Figure 4).

The mechanical properties of the material can be evaluated by DMTA, measured here in the torsion mode. The storage modulus is in the 1 GPa range (Table S1) at low temperature (below T_g_), which is characteristic of a cured epoxy material. The T_g_ was measured at the peak of the tan δ curve (Figure S8 and Table 2). For photocationic materials, one should expect that the T_g_ is close to the temperature at which they are synthesized, as captured in the TTT cure diagram [26]. In short, if the synthesis temperature polymerization is considerably lower than T_g_, the diffusion of polymer chains stop, preventing them from reacting in between them (vitrification, as observed for pure LDO). Interestingly, the determination of the T_g_ by DSC (during a second heating ramp) was found to yield higher values (80 °C instead of 21 °C for LDO/ESO 50/50), indicating that post-curing is possible upon heating the sample. Finally, it should be noted that materials that are richer in LDO have higher T_g_ and storage moduli, which can be explained by the high rigidity of the limonene backbone in comparison to the long and flexible alkyl chains of the vegetable oils.

### 3.3. Stereolithographic (SLA) 3D Printing Application

A standard commercial SLA printer was used to assess the possibility of using LDO in 3D-printing formulations. The schematic description of the stereolithographic printer is shown in Figure S9. The incident UV laser light (λ = 405 nm) emerges from the bottom of the platform, and the object is 3D printed from top to bottom by successive layer addition. Commonly available 3D printers are equipped with lasers emitting in the visible part of the electromagnetic spectra, where most photoacid generators do not absorb. To circumvent this limitation, a free-radical cationic polymerization approach using a radical photoinitiator absorbing at ~400 nm in addition to our photoacid generator was tested. Under these conditions, visible light is absorbed by the radical photoinitiator which in turn creates free radicals but also reactive cations through a redox mechanism involving the photoacid generator [27]. LDO is still the monomer of interest for the cationic part, and for the free-radical part, the focus is on a derivate of soybean oil: acrylated epoxidized soybean oil (AESO). Commercial AESO are formed upon reacting acrylic acid with ESO, leading to a vegetable oil in which two to four epoxides are converted in acrylates. As a monomer, AESO is both able to react via cationic ring-opening of the epoxide as well as via radical insertion polymerization. Formulations based on AESO possess inherent biodegradability but coatings based solely on AESO suffer from poor mechanical properties [28,29]. In our case, this behaviour was corrected by raising the crosslinking density by adding trimethylolpropane triacrylate (TMPTA) to our LDO/AESO mixture. The radical photoinitiator, phenylbis(2,4,6-trimethylbenzoyl) phosphine oxide (BAPO), was selected as it was reported to be an excellent initiator for AESO-based formulations [30]. As in any thermosets, the careful selection of the resin allows one to target the thermal and mechanical properties desired. With that mindset, we explored the properties of different ratios of LDO/AESO/TMPTA, as shown in Table 3 and Figure S11.

**Figure 5:**
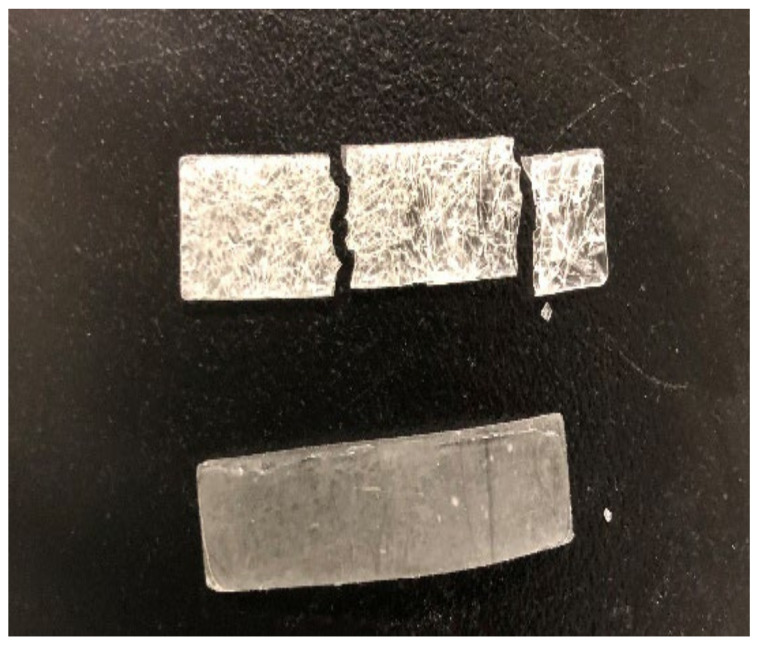
3D printed resins LDO/AESO/TMPTA 18/66/16 wt% (0.5%wt of BAPO); top: without PAG; bottom: with 0.5%wt of PAG.

To show the importance of the combination of radical and cationic system, three formulations made from LDO/AESO/TMPTA were photopolymerized. The first one, containing only BAPO as photoiniator, yielded a very brittle, solid sample. The second sample, containing only PAG, did not polymerize at all in the 3D printer. The third one, containing both BAPO and PAG, photopolymerized easily at ambient temperature to yield a solid, crack-free sample (Figure 5 and Figure S10). These results underscore the important synergy between both photoinitiators, which is critical for the correct polymerization of our LDO/AESO/TMPTA formulation.

When using only BAPO, the radical polymerization is very rapid, thus each photopolymerized pixel hardens before the next layer of material is deposited, approx. 20 s later, leading to a lack of cohesion between layers. The cationic photopolymerization is slower, and this second polymerization mechanism is necessary to provide cohesion between layers. This phenomenon is captured when monitoring the conversion of the epoxides (cationic pathway) and acrylates (radical pathway) by FTIR spectroscopy, using the bands at 760 cm^−1^ (C–O–C) and 1630 cm^−1^ (C=C) respectively. The conversion was determined approximately 1 h after the completion of the 3D printing (Table 3). The conversion of the acrylate is always around 60%, irrespective of the employed formulation. Due to the short free-radical life-time, the polymerization stops nearly immediately after the laser illumination stops. By contrast, the complete cationic polymerization of the LDO/ESO mix should take several hours once it is triggered, based on our results on cationic photopolymerization. For the epoxides, conversion measurement should be interpreted with care, as different positions in the sample will experience different irradiation conditions and will polymerize for different duration, by virtue of the 3D-printing process which is asynchronous. Importantly, immediately after 3D printing, the samples were found to be dry to the touch and devoid of residual unreacted monomers (no dissolution in methanol). The conversion of the epoxide (by cationic process) was found to increase as the proportion of acrylate increased (Table 3). As the radical process is dominating during the initial polymerization times, an increase in the mass ratio of acrylates in the mixtures is accompanied by a larger exotherm during photopolymerization. The heat generated by such a reaction could contribute to the observed synergy between the concomitant radical and cationic polymerizations. Interestingly, a post-curing for 30 min at 80 °C yielded a material in which not only were epoxy functionalities converted by the presence of the residual acid catalyst, but this also gave rise to an almost complete conversion of the acrylate groups (Table 3 and Figure S12).

Remarkably, swelling tests indicate that the samples are fully crosslinked, even in the absence of post-curing. From these swelling tests, it is found that the crosslinking density increases as the proportion of acrylate increases, once again suggesting that the radical polymerization is faster than the cationic one (Table S2).

**Figure 6:**
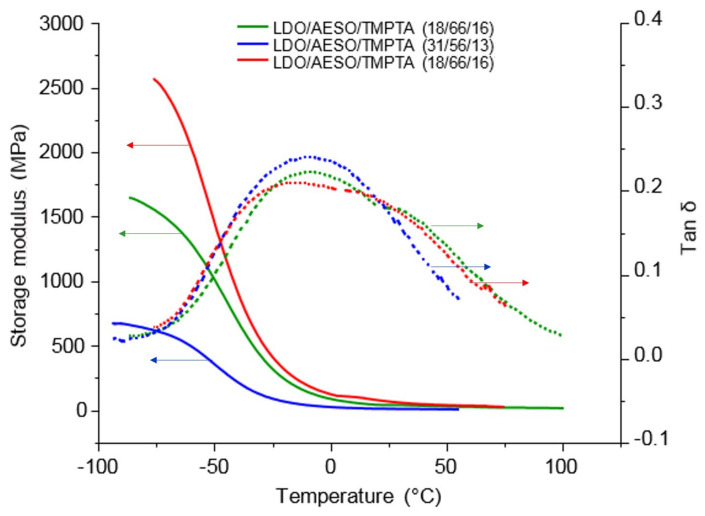
DMTA curves of LDO/AESO/TMPTA ratio (wt%) after 3D printing. Full lines show storage modulus curves and dash lines show tan δ curves.

The mechanical properties of 3D-printed dumbbells were analyzed by DMTA (Figure 6). Only one broad glass transition temperature is visible, suggesting that the two networks (formed via radical and cationic polymerizations) are interpenetrated, and no phase separation occurred in this dual curing system. Although the onset of the T_g_ is low (Table 3), the tan δ peak has a maximum which spans over 50 °C, resulting in objects that are hard at ambient temperature. The hardness of these objects is also shown by the Shore hardness measurement (Shore A = 93, which corresponds to the hardness of a semi-rigid plastic such as a plastic shoe heel or a shopping cart wheel).

In order to demonstrate the application of the LDO/AESO/TMPTA formulation (18/66/16) for SLA 3D printing, a small Eiffel tower was printed (Figure 1), using similar exposure parameters as those recommended for the commercial (non-renewable) formulation. This formulation was further characterized for a potential SLA application [31]. Under a 405 nm illumination, its critical energy and depth of penetration were found to be 28 mJ/cm^2^ and 72 μm. Remarkably, the biobased content of this formulation (LDO, TMPTA and acrylated ESO) was as high as 85%. Thus, this example provides a first proof of principle that biobased LDO and acrylated ESO are valuable biobased components for 3D-printed formulations.

## 4. Conclusions

In this article, we first examined the photocationic polymerization of limonene dioxide, and demonstrated that the vitrification phenomenon prevents this epoxy monomer to photopolymerize and to form continuous solids free of cracks. By adding epoxidized vegetable oils, the quantitative photocationic polymerization of limonene dioxide becomes possible at room temperature, to lead to the formation of crack-free, highly crosslinked, dry solids. Such photocationic formulation is, however, not suitable for stereolithographic 3D printing because its drying time is too slow. However, the acrylated epoxidized vegetable oil can photopolymerize rapidly via a radical mechanism, and then can more slowly copolymerize with limonene dioxide via a cationic mechanism. Such dual polymerization kinetics is ideal for the stereolithographic 3D process. Thus, a highly biosourced (up to 85%) formulation was developed and successfully used in a commercial stereolithographic 3D printer. These preliminary results demonstrate that limonene dioxide in combination with epoxidized vegetable oils could serve as key components in the elaboration of biobased stereolithographic 3D-printing formulations.

## Figures and Tables

**Table 1:** Polymerization of LDO/epoxidized oil with 1% of PAG (irradiation for 1 min, 60% relative humidity).

Oil	LDO/Oil wt%	Conversion Endocyclic Epoxide (%)	Conversion Exocyclic Epoxide (%)	Conversion Epox. Oil (%)	Drying Time (h)
none	100/0	92	46		1.5
ESO	60/40	>95	90	73	14
ESO	50/50	>95	>95	56	19
ESO	40/60	>95	>95	61	22
ESO	30/70	>95	>95	63	24
ELO	60/40	>95	>95	73	12
ELO	50/50	>95	>95	76	17
ELO	40/60	>95	88	58	21
ELO	30/70	>95	93	47	23

**Table 2:** Swelling ratio in methanol for the LDO/ESO and LDO/ELO polymers (1% of PAG, irradiation for 1 min, 60% relative humidity).

LDO/ESO wt%	Swelling in MeOH (%)	T_g_ by DMTA (°C)	LDO/ELO wt%	Swelling in MeOH (%)	T_g_ by DMTA (°C)
100/0	Dissolved		100/0	Dissolved	
60/40	38.5	-	60/40	11.5	40
50/50	11.1	21	50/50	4.1	32
40/60	8.4	22	40/60	2.4	31
30/70	5.7	17	30/70	1.3	26

**Table 3:** Composition and principal characteristics of the formulations used for 3D printing (all formulations initiated by 0.5 wt% PAG and 0.5 wt% BAPO, room temperature, relative humidity = 60%, λ = 405 nm).

LDO/AESO/TMTPA wt%	% Conversion Epoxide (760 cm^−1^)	% Conversion Acrylate (1630 cm^−1^)	T_g_ by DMTA (°C)
53/38/9	32	59	-
31/56/13	45	60	−8
18/66/16	66	62	−8
18/66/16^1^	75	95	−12

Curing for 30 min at 80 °C.

## Data Availability

Data are contained within the article.

## Supplementary Materials

The following supporting information can be downloaded at: https://www.mdpi.com/article/10.3390/polym16070965/s1, Figures S1–S7: FTIR spectra; Figure S8: Tan δ curves by DMTA; Figure S9: Schematic principle of stereolithography; Figure S10: Image by optical microscope of the 3D-printed resins; (a) without PAG; (b) with 0.5%wt of PAG; Figures S11 and S12: FTIR spectrum of mixtures before and after 3D printing; Table S1: storage and loss moduli of various formulations. Table S2: swelling ratios.
