# Supplementary material for: Photopolymerization of Limonene Dioxide and Vegetable Oils as Biobased 3D-Printing Stereolithographic Formulation
Source: Polymers (Basel). 2024 Apr 2;16(7):965. doi: 10.3390/polym16070965 (PMC11013986; doi:10.3390/polym16070965)
Supplement: Supplementary file 1 [file polymers-16-00965-s001.zip › polymers-2900825-supplementary.pdf]

Supplementary Materials

Photopolymerization of Limonene Dioxide and Vegetable Oils as Biobased 3D-Printing Stereolithographic Formulation

Megane Clerget[1,2], Eric Gagnon[2]*, Jerome P. Claverie[1]*

1: Université de Sherbrooke 2500 Boul. De l'Université, Sherbrooke, Qc, Canada, J1K 2R1

2: Soprema Canada, 1688 rue Jean Berchmans Michaud, Drummondville, Qc, Canada, J2C 8E9

Figures

Figure S 1 FTIR spectrum of the LDO/PAG mixture depending on the isomers cis-trans of LDO used (PAG = 0.1 wt%, 1 minute irradiation, 60% relative humidity). 3
Figure S 2 FTIR spectrum of LDO-ELO (50 wt%) mixture before and after photocationic polymerization (PAG = 1 wt%, 1 minute irradiation, 60% relative humidity). 4
Figure S 3 FTIR spectrum of LDO/PAG mixture with variation of the PAG concentration (1 minute irradiation, 60% relative humidity). 4
Figure S 4 Sample of LDO/PAG mixture, polymerized at ambient temperature (PAG = 0.1 wt%, 1 minute irradiation, 60% relative humidity). 5
Figure S 5 FTIR spectrum of LDO/PAG mixture with variation of irradiation time (PAG = 0.1 wt%, 60% relative humidity). 5
Figure S 6 FTIR spectrum of LDO/PAG mixture with variation of humidity (PAG = 0.1 wt%, 1 minute irradiation). 6
Figure S 7 FTIR spectrum of (a) LDO/ESO and (b) LDO/ELO mixtures at different concentration (%wt) before and after photocationic polymerization (PAG = 1 wt%, 1 minute irradiation, 60% relative humidity). 6
Figure S 8 Tan δ curves by DMTA for the crosslinked epoxy films after photocationic polymerization. (a) LDO/ESO and (b) LDO/ELO mixture (PAG = 1 wt%, 1 minute irradiation, 60% relative humidity). 8
Figure S 9 Schematic principle of stereolithography. 8
Figure S 10 Picture by optical microscope (DPM 300 from BYK); 3D printed resins LDO/AESO/TMPTA 18/66/16 %wt (0,5%wt of BAPO); (a) without PAG; (b) with 0,5%wt of PAG. 9
Figure S 11 FTIR spectrum of LDO/AESO/TMPTA mixtures (wt%) before and after 3D printing (0.5 wt% BAPO, 0.5 wt% PAG). 10
Figure S 12 FTIR spectrum of LDO/AESO/TMPTA mixtures 18/66/16 %wt before 3D printing, after 3D printing, and with a thermal postcure. 11

Tables

Table S 1 Storage modulus, loss modulus, at different temperature, by DMTA for LDO/ESO and LDO/ELO mixtures at different concentration (%wt) after photocationic polymerization (PAG = 1 wt%, 1 minute irradiation, 60% relative humidity). 7
Table S 2 Swelling tests in methanol for the mixture LDO/AESO/TMPTA printed by stereolithography... 10

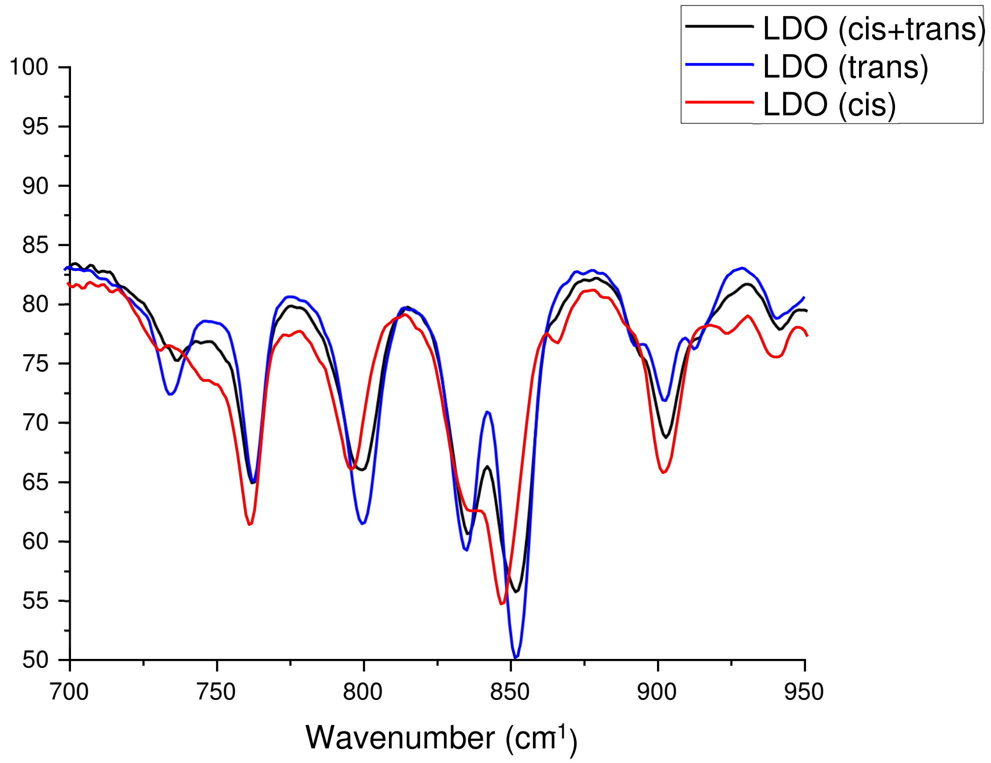

Figure S 1 FTIR spectrum of the LDO/PAG mixture depending on the isomers cis-trans of LDO used (PAG = 0.1 wt%, 1 minute irradiation, 60% relative humidity). The intensities are absolute (no normalization)

In order to compare FTIR spectra in between them, spectra were normalized by the band at 2950 cm^{-1} (see Figure S2). This is procedure is justified by the fact that the concentration of C-H bonds remains unchanged during the polymerization. Once normalized, the absolute y scale is arbitrary but samples become comparable to each other.

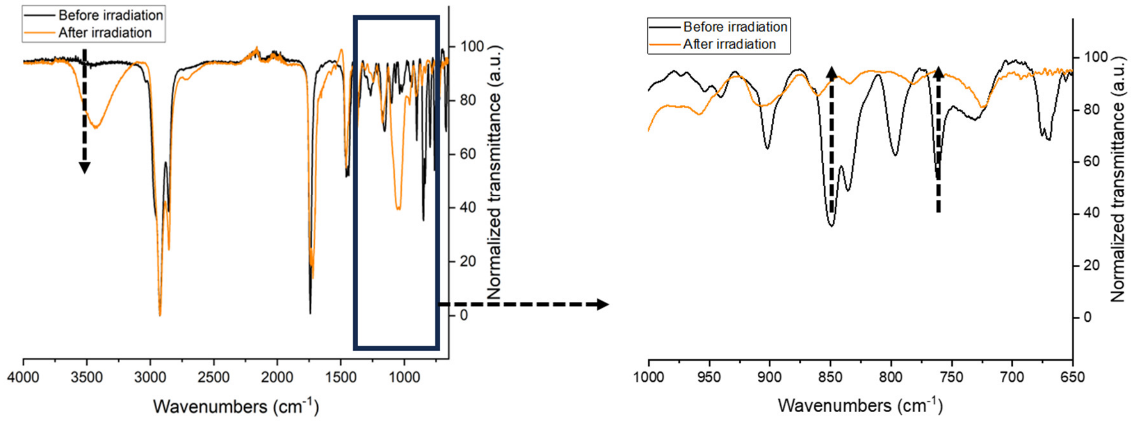

Figure S 2 FTIR spectrum of LDO-ELO (50 wt%) mixture before and after photocationic polymerization (PAG = 1 wt%, 1 minute irradiation, 60% relative humidity). The intensities have been normalized to the band at 2950 cm^{-1} (vertical scale is arbitrary).

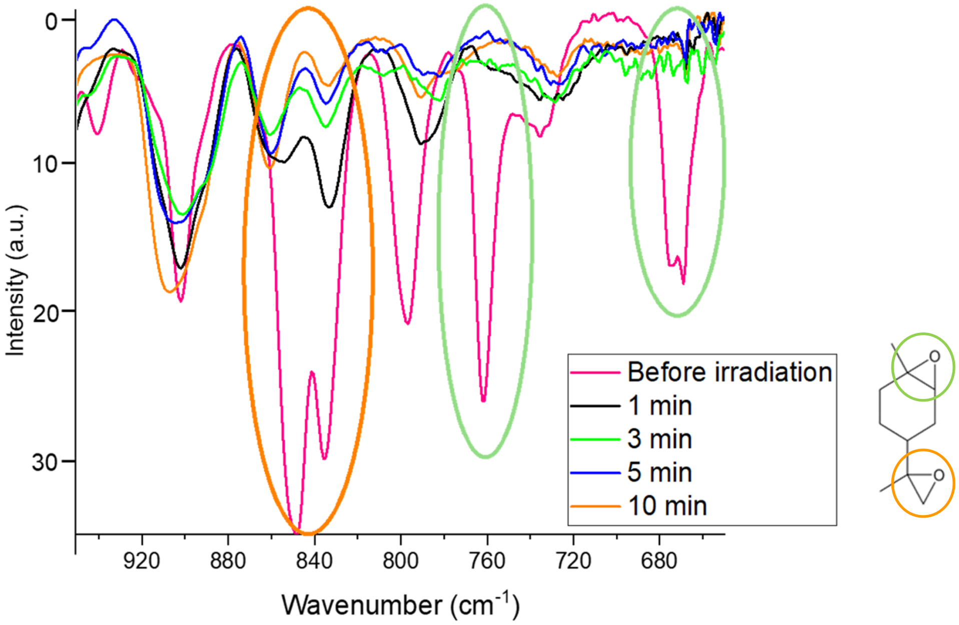

Figure S 3 FTIR spectrum of LDO/PAG mixture with variation of the PAG concentration (1 minute irradiation, 60% relative humidity). The intensities have been normalized to the band at 2950 cm^{-1} (vertical scale is arbitrary).

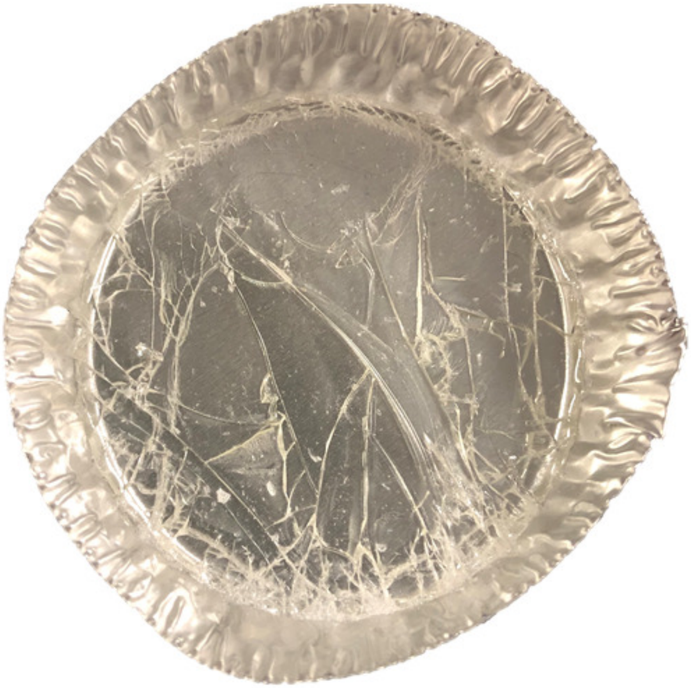

Figure S 4 Sample of LDO/PAG mixture in an aluminum dish, polymerized at ambient temperature (PAG = 0.1 wt%, 1 minute irradiation, 60% relative humidity).

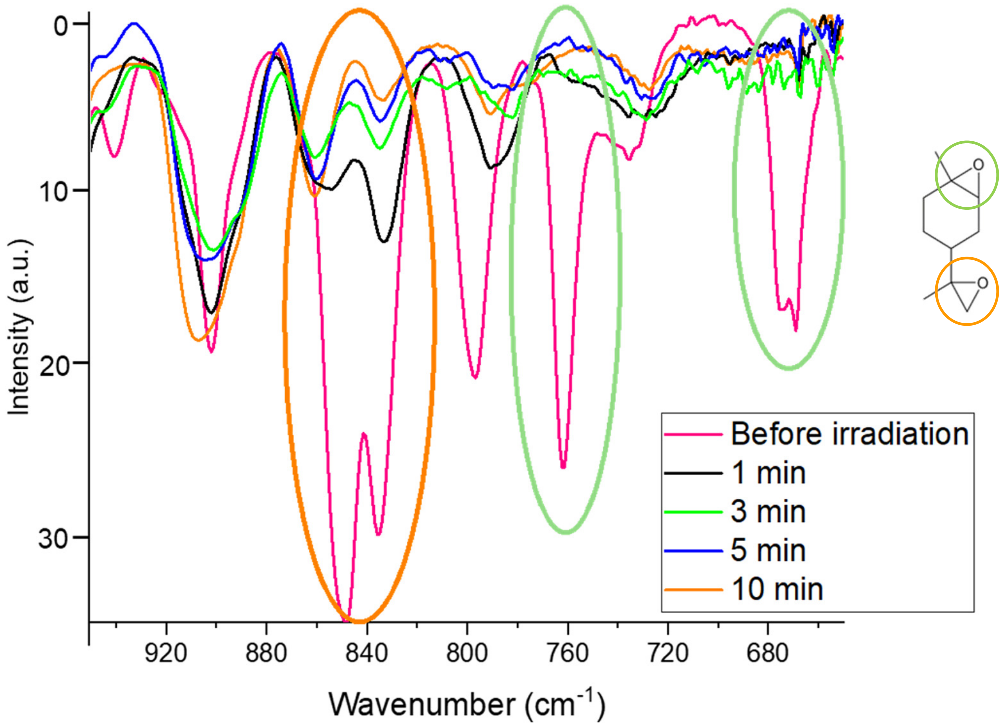

Figure S 5 FTIR spectrum of LDO/PAG mixture with variation of irradiation time (PAG = 0.1 wt%, 60% relative humidity). The intensities have been normalized to the band at 2950 cm^{-1} (vertical scale is arbirtrary).

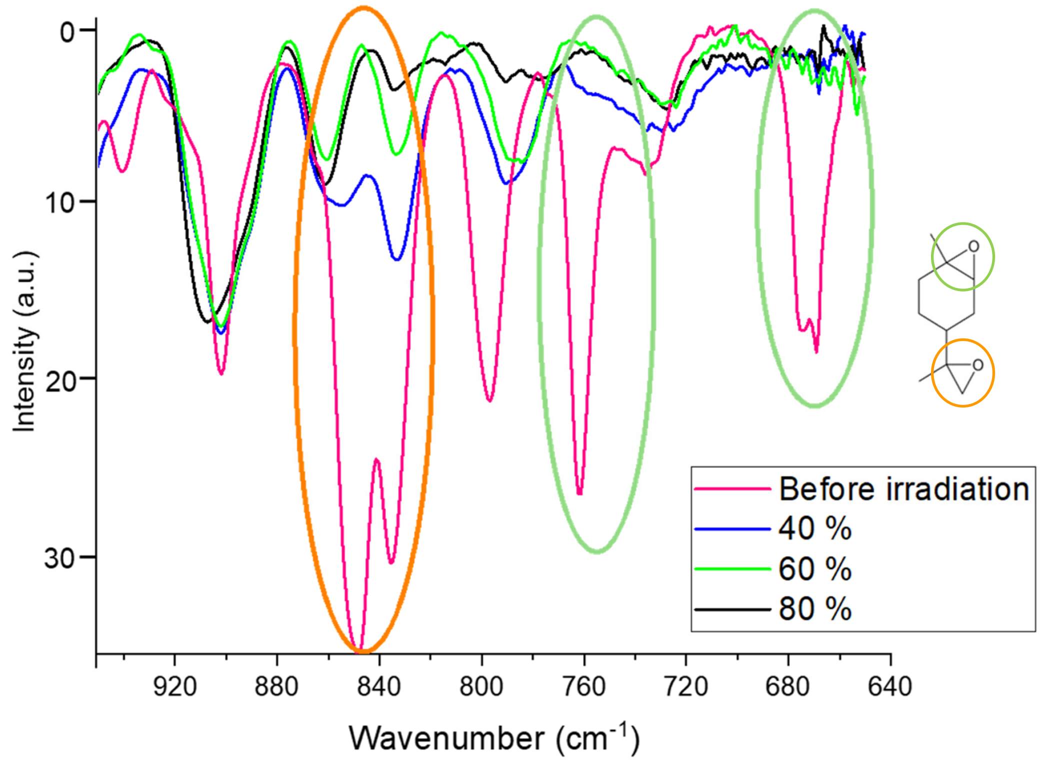

Figure S 6 FTIR spectrum of LDO/PAG mixture with variation of humidity (PAG = 0.1 wt%, 1 minute irradiation). The intensities have been normalized to the band at 2950 cm⁻¹ (vertical scale is arbirtrary).

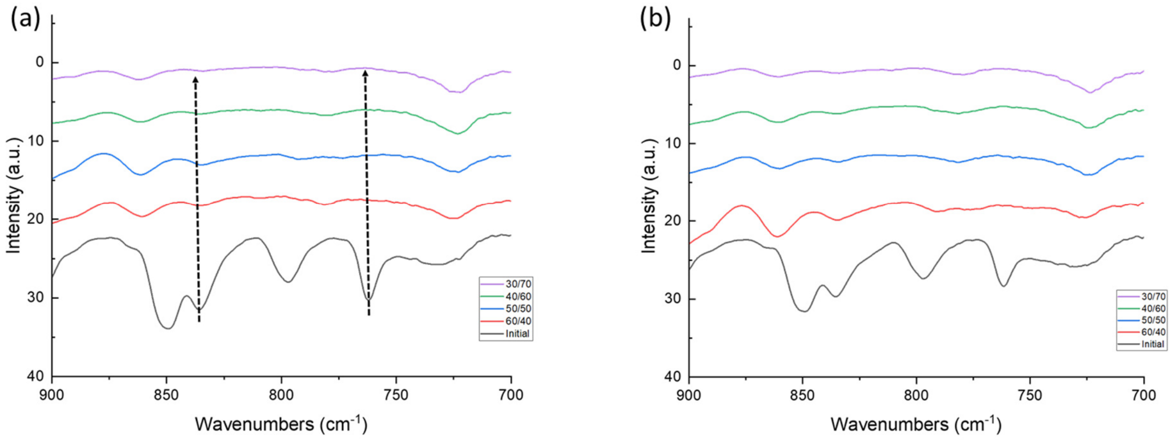

Figure S 7 FTIR spectrum of (a) LDO/ESO and (b) LDO/ELO mixtures at different concentrations (%wt) before and after photocationic polymerization (PAG = 1 wt%, 1 minute irradiation, 60% relative humidity). The intensities have been normalized to the band at 2950 cm⁻¹ (vertical scale is arbirtrary). Note that the spectra have been vertically offset for the sake of clarity.

Table S 1 Storage modulus, loss modulus, at different temperature, by DMTA for LDO/ESO and LDO/ELO mixtures at different concentration (%wt) after photocationic polymerization (PAG = 1 wt%, 1 minute irradiation, 60% relative humidity).

**Formulation**	**-30°C**		**0°C**		**25°C**	
	Storage modulus (MPa)	Loss modulus (MPa)	Storage modulus (MPa)	Loss modulus (MPa)	Storage modulus (MPa)	Loss modulus (MPa)
**LDO/ESO 30/70 (wt%)**	1244±20	62±1	176±14	102±1	2±1	2±1
**LDO/ESO 40/60 (wt%)**	1581±22	94±1	390±21	162±3	3±1	4±1
**LDO/ESO 50/50 (wt%)**	874±16	65±1	234±16	93±1	4±2	5±1
**LDO/ELO 30/70 (wt%)**	1070±18	84±2	840±26	87±1	71±13	47±6
**LDO/ELO 40/60 (wt%)**	1103±12	28±1	793±24	70±1	121±21	74±4
**LDO/ELO 50/50 (wt%)**	933±15	32±1	754±17	71±1	255±26	114±11

(a)

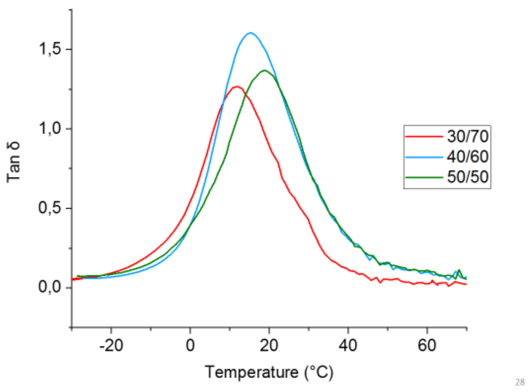

(b)

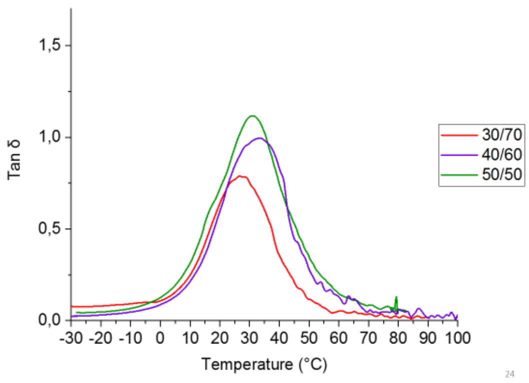

Figure S 8 Tan δ curves by DMTA for the crosslinked epoxy films after photocationic polymerization. (a) LDO/ESO and (b) LDO/ELO mixture (PAG = 1 wt%, 1 minute irradiation, 60% relative humidity).

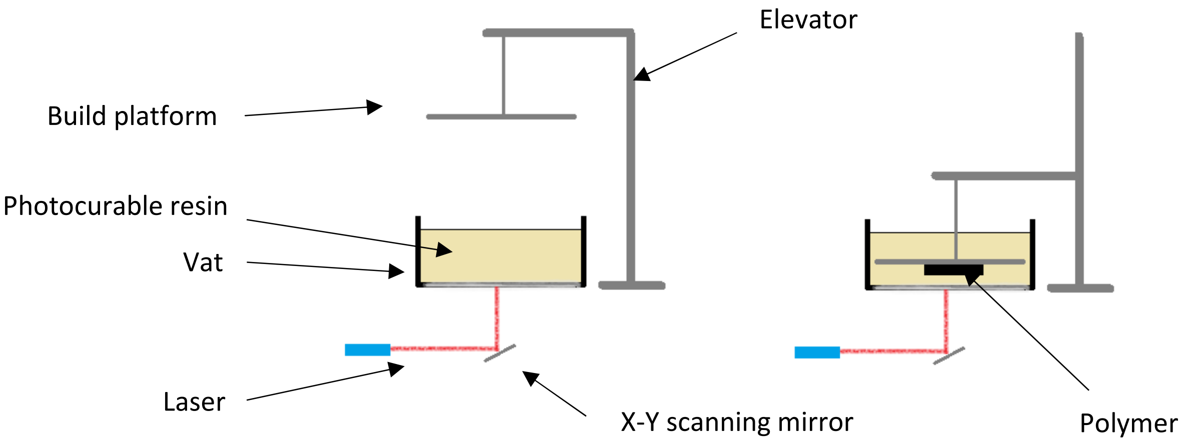

Figure S 9 Schematic principle of stereolithography.

(a)

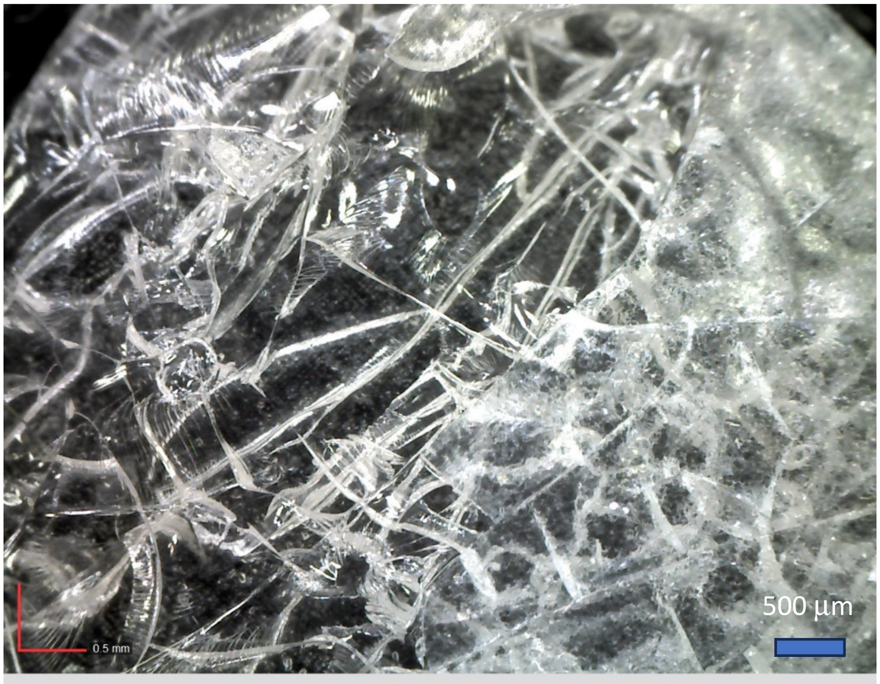

(b)

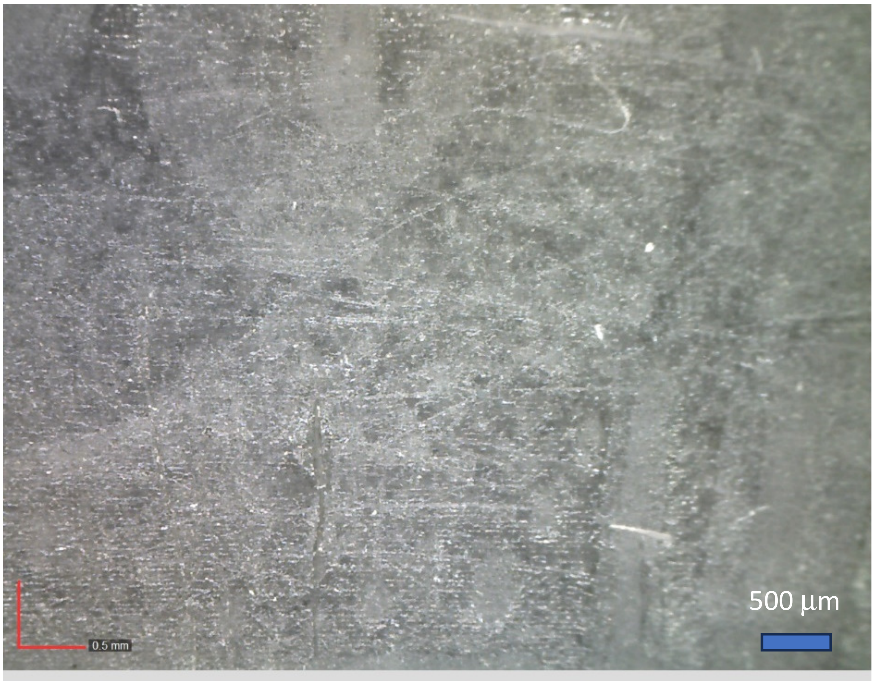

Figure S 10 Picture by optical microscope (DPM 300 from BYK); 3D printed resins LDO/AESO/TMPTA 18/66/16 %wt (0,5%wt of BAPO); (a) without PAG; (b) with 0,5%wt of PAG.

Table S 2 Swelling tests in methanol for the mixture LDO/AESO/TMPTA printed by stereolithography.

LDO/AESO/TMTPA (wt%)	Swelling in MeOH (%)
**53/38/9**	15.1
**31/56/13**	10.8
**18/66/16**	3.6
**18/66/16[1]**	2.4

1. curing for 30 minutes at 80°C.

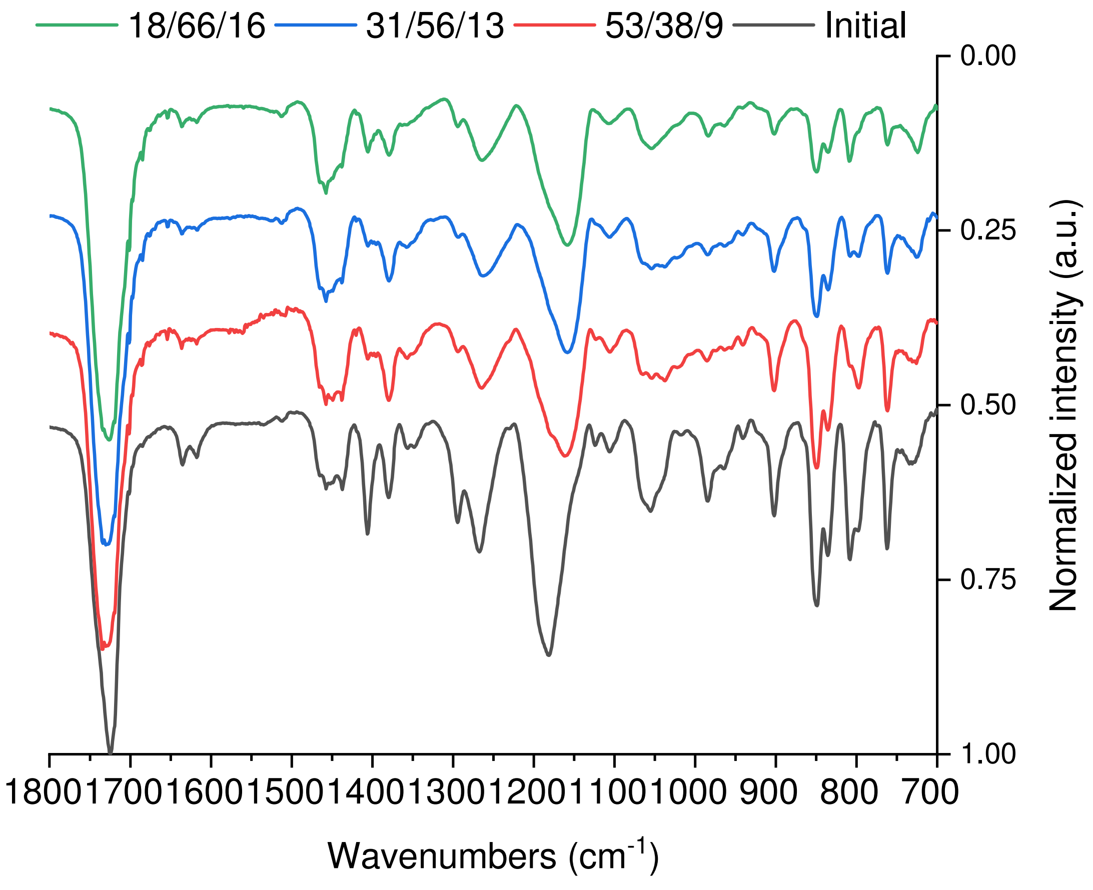

Figure S 11 FTIR spectrum of LDO/AESO/TMPTA mixtures (wt%) before and after 3D printing (0.5 wt% BAPO, 0.5 wt% PAG). The intensities have been normalized to the band at 2950 cm⁻¹ (vertical scale is arbirtrary). Note that the spectra have been vertically offset for the sake of clarity.

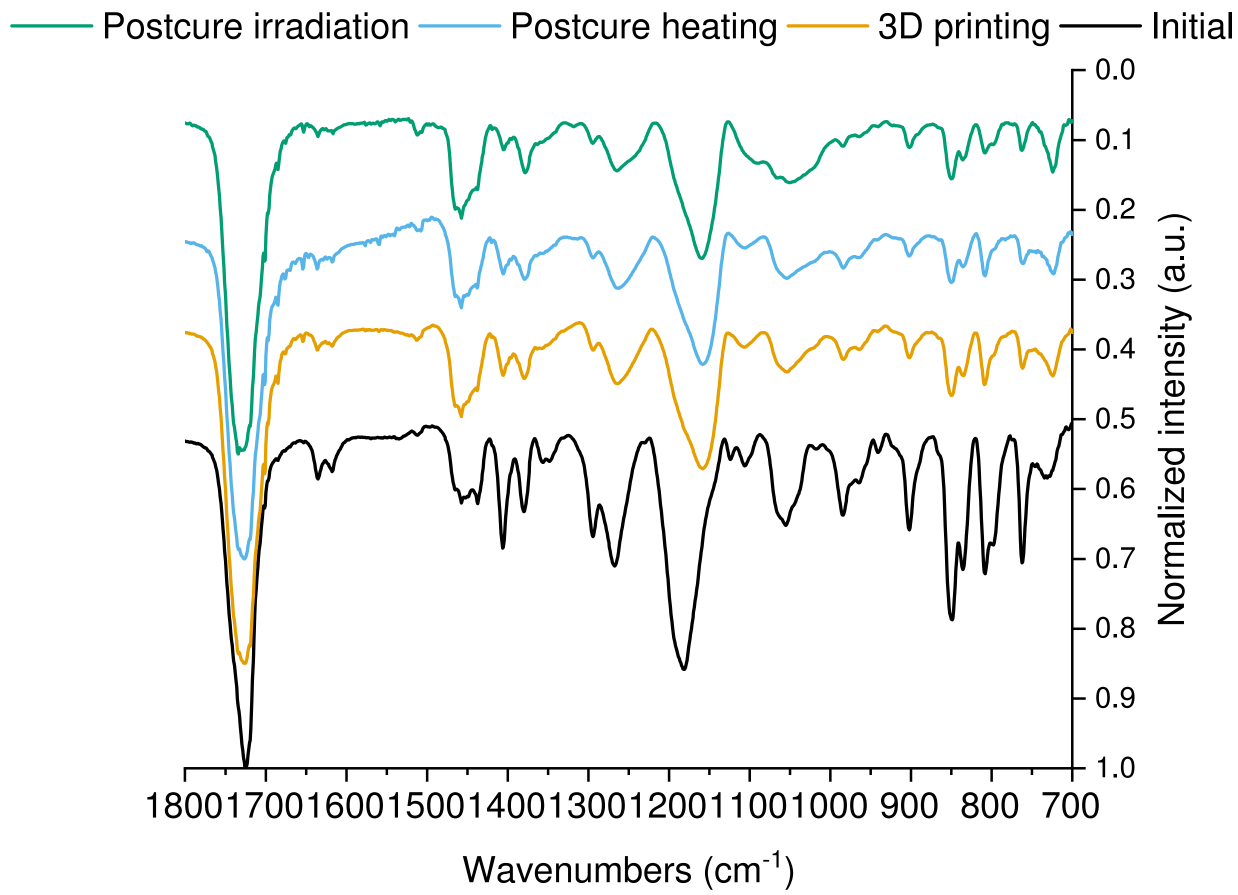

Figure S 12 FTIR spectrum of LDO/AESO/TMPTA mixtures 18/66/16 %wt before 3D printing, after 3D printing, and with a thermal postcure. The intensities have been normalized to the band at 2950 cm^{-1} (vertical scale is arbirtrary). Note that the spectra have been vertically offset for the sake of clarity.
